# Clinical study on resin composite and glass ionomer materials in II class restorations in permanent teeth

**DOI:** 10.4317/jced.57572

**Published:** 2021-02-01

**Authors:** Piotr Rożniatowski, Emil Korporowicz, Dariusz Gozdowski, Dorota Olczak-Kowalczyk

**Affiliations:** 1 Department of Paediatric Dentistry, Medical University of Warsaw, Poland; 2 Department of Experimental Statistics and Bioinformatics, Warsaw University of Life Sciences, Poland

## Abstract

**Background:**

Glass ionomer cements (GIC) used for restoration of missing dental structures have high biocompatibility and remineralization potential. However, low mechanical resistance excludes their use for long-term restorations of extensive lesions, particularly on approximal surfaces in permanent dentition. GIC with increased viscosity have much better physical properties, which involves better bonding and wear resistance, so they can be considered as an alternative to composite resin materials. The aim of this study was to perform a clinical and radiological assessment of restorations in permanent teeth, made with an increased viscosity GIC - Equia Fil (Ivoclar Vivadent) with Equia Coat (Ivoclar Vivadent) and composite material - Tetric EvoCeram (Ivoclar Vivadent) in young patients.

**Material and Methods:**

A total of 100 cavities on approximal surfaces were filled with the composite material or GIC in 49 patients aged from 12.08 to 19.58 years. During control examinations, the condition of each restoration was assessed with criteria acc. to Hickel et al. Bitewing radiographs had been taken before fillings were placed and after 12 and 24 months.

**Results:**

After two years of observations, two GIC restorations were replaced due to loss of retention and staining. The other 96 restorations were given a satisfactory grade. The clinical efficacy of Equia Fil after 24 months was assessed at 95.83%, the Tetric EvoCeram at 100%. The difference was not statistically significant (*P*=0.145). When GIC was used, there was a higher risk of marginal adaptation deterioration and the occurrence of staining and erosion. Radiographic efficacy of the Equia Fil material for cavity restoration after 24 months was assessed at 93.75%, for the Tetric EvoCeram material at 100%. Differences were not significant statistically (*P*=0.073).

**Conclusions:**

Tetric EvoCeram and Equia Fil used for the restoration of approximal lesions in premolars and permanent molars have similar efficacy in a 2-year period of observation.

** Key words:**Dental restoration, permanent, composite resins, glass ionomer cements, clinical study.

## Introduction

Composite materials are the most frequently used ones for direct restoration of missing dental structures on approximal surfaces of premolars and permanent molars (1-4). They are characterized by durability, hardness and wear-resistance comparable with enamel; they also have excellent polishability (5). However, they do not have the potential for remineralization of the partially demineralized dentine at the bottom of the cavity. They are also burdened with cytotoxicity, which can decrease treatment efficacy of caries profunda. Several researchers associate the occurrence of pulpal inflammatory reactions, as well as cellular apoptosis, with monomers that they release (6). It is particularly valid in newly erupted permanent teeth with higher permeability of dentine (thin layer of primary dentine, wide dentinal tubules). Glass ionomer cements have high biocompatibility and remineralization potential. They are readily used in pediatric dentistry for permanent restorations of lesions in deciduous teeth and for correction of minor defects in permanent teeth; they are also suiTable as temporary restorations of deep carious lesions, or when reduction of the risk of caries is necessary (5,7,8). Low mechanical resistance of standard glass ionomer cements excludes their use for long-term restorations of extensive lesions, particularly on approximal surfaces in the permanent dentition.

Glass ionomer cements with increased viscosity have much better physical properties, which involves better bonding, good wear resistance, low solubility in the oral environment and low sensitivity to moisture; they are also characterized by better aesthetics (9,10,11,12,13).

 Wear resistance and surface hardness, as well as aesthetics of the restoration, can additionally be increased by an application of a protective varnish with a nanofiller. As far as Equia Fil (Ivoclar Vivadent) GIC with increased viscosity is concerned, the application of Equia Coat (Ivoclar Vivadent) protective varnish can increase the material’s flexure resistance by 48% (14). This material is recommended for restoration of Class I cavities acc. to Black, as well as minor lesions on approximal surfaces. However, their application in extensive carious defects on approximal surfaces in newly erupted permanent teeth still requires sounder scientific basis (15,16).

The aim of this study was to perform a clinical and radiological assessment of the quality of restorations of approximal lesions in permanent teeth made with Equia Fil glass ionomer cement and Tetric EvoCeram (Ivoclar Vivadent) composite in adolescents and young adults. The effect of clinical parameters on the quality of fillings has also been examined.

## Material and Methods

Clinical and radiological examinations included baseline assessment and two reviews (I – after 12 months and II – after 24 months). The study also involved therapeutic intervention: making a restoration on the approximal surface of a molar or a premolar with a composite material (group A) or a GIC (group B). The examinations were carried out by two dental practitioners following training and calibration (Kappa coefficient: 0.89). The consent of the Bioethics Committee at the Medical University of Warsaw was obtained KB/157/2013.

The study subjects were selected from patients presenting at the Department of Paediatric Dentistry, Medical University of Warsaw. The inclusion criteria were as follows: age between 12-20 years, Class II carious lesion acc. to Black (code 4 or 5 acc. to ICDAS II; code D1, D2 or D3 acc. to Manji *et al.* for molars and premolars), written consent for participation in the study expressed by patients and/or parents/legal caregivers. The exclusion criteria included recurrent caries, symptoms of pulpitis in a tooth with a approximal defect (pain, improper response to pulp vitality testing), occlusal parafunction (bruxism), malocclusion or an on-going orthodontic treatment, history of chronic disease requiring specialist medical care, planned change of a place of residence within a year, absence of consent.

Clinical examination was carried out in a dental surgery by means of a WHO-621 periodontal probe (17). It included assessment of oral hygiene status by means of a simplified oral hygiene index (OHI-S) acc. to Greene and Vermillion (1964), condition of dentition – the presence of dental caries on all dental surfaces acc. to International Caries Detection and Assessment System (ICDAS-II), pulp vitality thermal test with ethyl chloride and electrical test (Sybron Endo Vitality Scanner) of a tooth to be treated and compared with its contralateral counterpart. At baseline, DMFT was calculated, with D being the value of ICDAS II 1 and 2 codes, with *P* – values of ICDAS II codes ≥3. During control examinations, additionally the condition of the restoration was assessed with criteria acc. to Hickel *et al.*, recommended by the World Dental Federation (FDI) (Table 1) (18) 

During review visits, the patients received dietary tips. Oral hygiene instructions were delivered together with a topical application of a fluoride varnish.

**Table 1:**
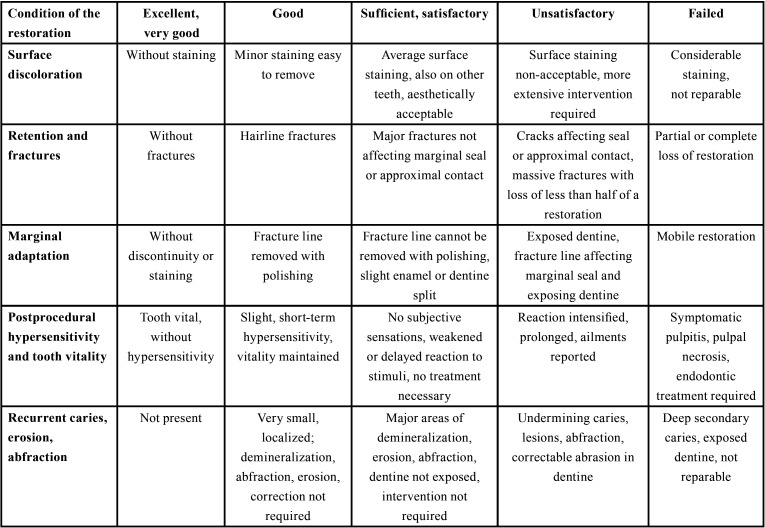
Evaluation criteria of individual characteristics of restorations acc. to Hickel *et al.*

Bitewing radiographs had been taken before fillings were placed and after 12 and 24 months. Depth of cavities in dentine on approximal surfaces was assessed acc. to Manji: D1 – radiolucency in 1/3 of the outer dentine, D2 – radiolucency in 1/3 of the central dentine, D3 – radiolucency reaching 1/3 of the inner dentine (19). Control radiographic examinations served to assess the presence of deficiency/excess of the material and radiolucency near the gingival wall of the tooth, which might indicate the presence of a marginal gap and thus secondary caries. It also served to determine the presence of horizontal and vertical bone defects in interdental spaces indicating marginal periodontitis, fracture of mineralized dental tissues and altered shape of the restoration. The occurrence of any of the above-listed manifestations qualified the restoration as unsatisfactory (19).

In accordance with the principles of randomized trials, selection was made with the use of block size 6 allocation for two types of intervention (A, B) assigned to tooth numbers (n) in the order of patients’ presentation in the surgery. If more than one tooth qualified for intervention in one patient, the order in which it was performed was determined by the location of a given tooth in the mouth: first, tooth 16, followed by 26, 36 and 46. Blinding of the type of intervention was obtained by placing the result of the draw in non-transparent envelopes. This form of allocation concealment made it possible to protect the randomization process and prevent access to the information about the group to which the given patient was allocated before inclusion in the study.

Class II lesions in premolars and molars were prepared according to generally accepted methods of cavity restoration with composite materials and GIC. A layer of partially demineralized dentine was left at the bottom of the carious lesion. In order to retain the shape of the tooth, 0.045 mm anatomic matrix strips were used; the operating area was isolated with cotton wool rolls (20,21).

 The materials were applied according to manufacturers’ instructions. Tetric EvoCeram with ExciteF (Ivoclar Vivadent) bonding agent (intervention A) was applied in layers (incremental technique), Equia Fil (intervention B) with a single-layer technique. Composite materials were prepared directly after placement. The final preparation of Equia Fil was undertaken 2.5 min after commencement of mixing. The surface was covered with Equia Coat and polymerized for 20 seconds.

The results of the study were subjected to statistical analysis by means of Statistica 12 (StatSoft) for Windows (Microsoft). For each calculation the significance level was set at p≤0.05. Data for statistical analysis was obtained from previously prepared Excel spreadsheets (Microsoft). Comparisons of mean values and clinical and radiological effectiveness of materials was performed with U Mann-Whitney and chi-square tests.

## Results

A total of 100 cavities on approximal surfaces were filled in 49 patients aged from 12.08 years to 19.58 years (mean 15.87±1.80 years), with mean OHI-S 1.32±0.54 and mean DMFT 11.25±5.01. Clinical parameters of patients qualified for the study and the number of restorations with Tetric EvoCeram and Equia Fil depending on the localization of treated teeth and depth of cavities are presented in Table 2. Depth of cavities, as assessed radiographically, is presented in Table 3. After 12 months, 47 participants presented for the first review (98 fillings). After 24 months, 45 patients were re-examined (96 fillings).

**Table 2:**
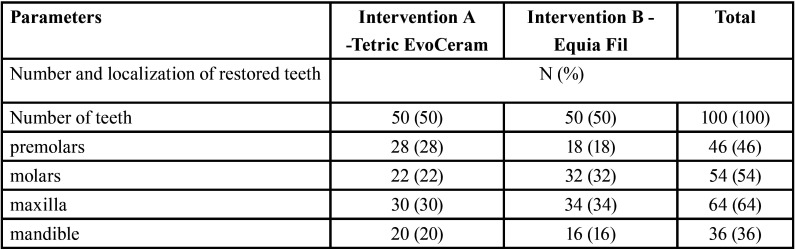
Clinical parameters of the examined patients and the type of restoration on approximal surfaces.

**Table 3:**
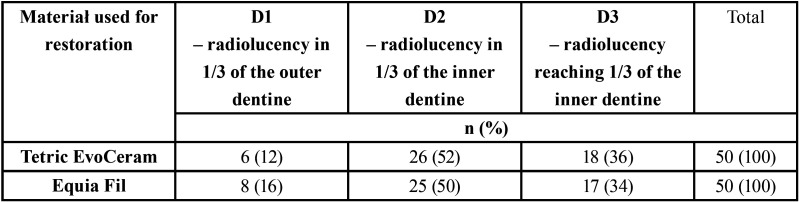
Depth of lesions filled with Tetric EvoCeram and Equia Fil – radiographic evaluation.

During the first control examination after 12 months, 50 teeth filled with Equia Fil and 48 teeth filled with Tetric EvoCeram were reviewed. The unsatisfactory grade (score 5) was given to two GIC restorations. In one case, the patient presented with a temporary filling placed by an independent GDP because the original GIC filling had been lost. The other restoration covered by the study also received the score of 5 due to staining and loss of retention, and the score of 4 because the restoration manifested signs of erosion.

All 96 restorations reviewed during the control examination after 24 months were given a satisfactory grade. The two Equia Fil restorations, which previously had received an unsatisfactory grade, were not taken into consideration. Postprocedural hypersensitivity was not recorded. Pulpal vitality, when examined by appropriate tests, was normal.

The analysis of Spearman correlation failed to confirm any association between the condition of the restorations and a specific type of tooth under treatment, its position in the maxilla or the mandible. When the U Mann-Whitney test was used for comparison of mean values of the studied materials (p≤0.05) the statistically significant differences were observed for such criteria as: the general condition of the restoration, staining, marginal adaptation and erosion (Table 4).

**Table 4:**
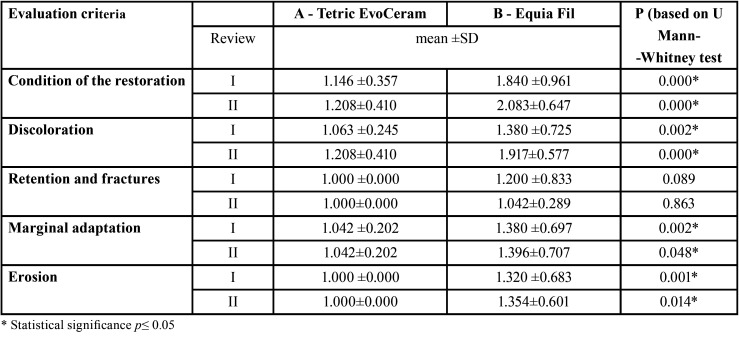
Comparison of mean values of the examined materials after 12 and 24 months (review I and II).

The clinical efficacy of the Equia Fil material used for the restoration of approximal lesions in molars and premolars after 12 months was assessed as 95.83%; for the Tetric EvoCeram material at 100%. The demonstrated difference was not statistically significant *P*=0.145. After 24 months, no restorations were assessed as unsatisfactory, thus the efficacy of both materials was identical, similar to the review examination performed after 12 months.

Based on radiographic examination, three fillings (6%) made with the Equia Fil material were assessed as unsatisfactory after 12 months. In one case, altered shape of the restoration in the vicinity of the approximal contact was observed; in two cases, there was a suspicion of recurrent caries/presence of a marginal gap. For composite restorations, no complications were noted radiographically. At 24-month review, all restorations were radiographically considered satisfactory. Radiographic efficacy of the Equia Fil material for cavity restoration after 12 months was assessed at 93.75%, for the Tetric EvoCeram material at 100%. After 24 months, these values remained the same. Differences were not significant statistically (*P*=0.073).

When both clinical and radiological features of the restorations are concerned, the efficacy for Equia Fil and Tetric EvoCeram was 91.67% and 100%, respectively after 12 and 24 months of observation. The difference was statistically significant (*P*=0.037).

## Discussion

The properties of composite materials, such as amalgam-like durability, ease of application and high acceptance by patients, are an argument for their widespread use in the clinical practice when it comes to restoration of approximal lesions in permanent teeth (1-4). Their clinical suitability has been confirmed by Pallesen *et al.* (21). These researchers evaluated the efficacy of composite materials used for restorations of Class I and II lesions in permanent teeth of children and adolescents (mean age 13.7 years). A total of 4355 restorations were evaluated (Class I cavities – 49%, Class II – 41.7%, multi-surface lesions – 9.3%). In the course of 8 years of observations, 406 restorations had been replaced and 125 required correction. An overall efficacy of composite materials was assessed at 97.7% after a year, 92.8% – after 3 years, 87.5% – after 5 years, and 84.3% – after 8 years. After 5 years, the efficacy of the tested materials used for Class I cavities was higher (91%) in comparison with Class II cavities (85%) and multi-surface ones (81%). The authors, however, did not present results of a two-year observation of Class II fillings. The overall one-year assessment of all restorations (97.7%) was similar to the one obtained by the authors of the present study in a comparable observation period.

When studies were being reviewed for the purpose of the present study, no clinical failure had been reported concerning the Tetric EvoCeram composite material. Cetin *et al.* utilized the so-called modified Ryge scale for the assessment of three different composite materials used for the restoration of Class I and II cavities acc. to Black in patients aged 20-28 years. The authors examined the Filtek Supreme XT, Aelite Aesthetic and Tetric EvoCeram materials in a year-long observation. After 12 months, 100% efficacy was noted for all of these materials. Retention and gingival adaptation had the highest Alpha score of 100% for each material. In the “surface smoothness and marginal adaptation” category Tetric EvoCeram and Aelite Aesthetic materials obtained 95% Alpha score and 5% Bravo score, which indicates accepTable condition of restorations. In the “surface smoothness” and “marginal staining” categories the lowest score was given to Filtek Supreme XT (Alpha score of 80% and 85%, and Bravo score of 20% and 25%, respectively). All the other criteria received Alpha scores (100%) on the scale used by the authors No statistically significant differences were observed between the materials. These results seem similar to the ones obtained in the present study after one year of observations, yet direct comparison is not possible. In the studies by Cetin *et al.*, sixty fillings in total were evaluated (twenty each for three different materials). Filtek Supreme XT was used for eleven Class I and nine Class II fillings, Tetric EvoCeram for twelve Class I and eight Class II fillings, and Aelite Aesthetic for fourteen and six fillings, respectively (22).

Comparable results were obtained by Schoch *et al.* who used Tetric Ceram for the restoration of six Class I lesions and nineteen Class II lesions in permanent teeth of patients aged 19-41 years. After one year, 96% of restorations were scored as Alpha in all evaluation criteria. One filling (4%) received a Bravo score due to the presence of marginal staining; however, the authors failed to mention the class of the restoration in question. All Class I and II restorations were assessed as satisfactory at the 12-month review. The authors, however, failed to review the restorations after 24 months (23).

Unlike composite restorations, when Equia Fil glass ionomer restorations were reviewed after 24 months, 4% of failures were noted. In one case, discoloration, fracture and surface erosion were present; in another case, there was total loss of retention. Gurgan *et al.*, who assessed Class II Equia Fil restorations in permanent teeth of patients aged 15-37 years, did not report such failures (24). These authors reviewed thirty fillings, which were all assessed as accepTable. Only 6.9% of fillings were stained and in 13.8% of cases degradation of the marginal seal was noted. In the present study, at 12- and 24-months review, 28% and 79% Equia Fil discolored restorations respectively were observed, which received accepTable score; 2% of restorations were assessed as unaccepTable. Gurgan *et al.* did not conduct a 2-year review, and so it is impossible to undertake any comparative analysis with the results of the present study. However, 26% of their restorations had a score higher than 1 (acc. to Hickel *et al.*) when marginal adaptation is concerned, and this outcome is comparable with the one obtained in the present study for the same study period.

 Scholtanus *et al.* (25) reported similar efficacy of the Fuji IX GP Extra material (renamed as Equia Fil in some countries) for Class II restorations.

In a retrospective study, they assessed 116 fillings, which were coated with a protective varnish. In the 18-month observation period they did not observe any failures. No information, however, was provided concerning the patients’ age or the time that had elapsed between the eruption of a tooth and the moment of observation. Teeth with both primary and recurrent caries were included in the study.

Fried *et al.* in a retrospective study conducted in six German clinics reported no loss of restorations. Here, 125 Class II cavities had been restored with Fuji IX GP Extra in 43 patients in permanent teeth in a 2-year observation period (20). In this case, similarly to the other previously cited authors, patients’ age was not given. In 44% of the restorations, loss of approximal contact was observed; however, for the 35% of the restorations it amounted to a slight change of the restoration’s volume at the approximal surface, not resulting in total loss of contact. Total loss of approximal contact was observed in five restorations (4%), which were qualified for replacement. In the present study, the altered shape of the restoration in the vicinity of a contact point was observed in one tooth only, basing on a radiographic examination carried out after 12 months. Fried *et al.* reported the presence of surface roughness in seven Class II restorations and loss of marginal seal only in one case (0.8%). Because of these findings, nine restorations (7.2%) were qualified for replacement after two years of observation. The authors attributed the altered shape of the restoration in the vicinity of the contact point to the absence of coating with protective varnish. This view is shared by other researchers who noted increased wear of the material in comparison with the enamel in 37% of the restorations not protected with varnish and 28% that had been coated (26). The observed difference in the wear of the material was not statistically significant. Scholtanus reported similar opinions concerning the result of his studies. The author claimed that the contact point and the edge of the restoration gingivally are places that are not accessed by the liquid protective varnish (25). It is likely that the absence of varnish in these areas resulted in loss of approximal contact and the presence of a marginal gap gingivally in two fillings in the present study. It is recommended that the Equia Fil material be used with a dedicated protective varnish with a nanofiller, the application of which increases surface hardness, the material’s aesthetics and wear resistance (20,24,26,27). Lohbauer *et al.* (28) confirmed increased wear resistance of the Fuji IX GP Extra material if it is coated with varnish containing a nanofiller. However, an adverse effect of the varnish was documented since it impedes fluoride release from glass ionomer cement to the oral environment (29).

Rutar *et al.* conducted a three-year observation of 129 Fuji IX GP Extra restorations (Class I – 56, Class II – 73); after one year the material was assessed at 96%, after three years at 94% (30). Considering marginal staining, 90% of Class I restorations and 100% of Class II restorations were deemed accepTable. In the marginal adaptation category, these values were 100% and 93%, respectively. No recurrent caries was observed. Deciduous second molars had been filled and evaluated with Ryge modified criteria. The authors claimed that the high success rate could be attributed to the minimally invasive cavity preparation and to little exposure to occlusal forces. In the present study, no correlation was defined between the size of the restoration and their assessment after 24 months. However, photographic documentation confirms that the GIC restoration, which failed, was large.

In conclusion, Tetric EvoCeram composite material and Equia Fil glass ionomer used for the restoration of approximal lesions in premolars and permanent molars of adolescents have similar clinical and radiological efficacy in a 2-year period of observation. When GIC is used, there is a higher risk of marginal adaptation deterioration and the occurrence of staining and erosion.
